# The association between the outcomes of extraperitoneal laparoscopic radical prostatectomy and the anthropometric measurements of the prostate by magnetic resonance imaging

**DOI:** 10.1590/S1677-5538.IBJU.2017.0260

**Published:** 2018

**Authors:** Sompol Permpongkosol, Supanun Aramay, Thawanrat Vattanakul, Sith Phongkitkarun

**Affiliations:** 1 Division of Urology, Department of Surgery, Faculty of Medicine, Ramathibodi Hospital, Mahidol University, Bangkok 10400, Thailand; 2 Department of Diagnostic and Therapeutic Radiology, Faculty of Medicine, Ramathibodi Hospital, Mahidol University, Bangkok, Thailand

**Keywords:** Prostatectomy, Prostatic Neoplasms, Magnetic Resonance Imaging, Laparoscopy

## Abstract

**Introduction and objective:**

To determine the association between the anthropometric measurements by magnetic resonance imaging (MRI) and perioperative outcomes of extraperitoneal laparoscopic radical prostatectomy (ELRP).

**Materials and Methods:**

From 2008 to June 2016, 86 patients underwent preoperative MRI prior to undergoing ELRP for localized prostate cancer. We analyzed the associations between anthropometric measurements of MRI and the perioperative outcomes of patients who underwent ELRP.

**Results:**

The mean patient age was 69.61±8.30 years. The medians of operating time and blood loss were 2.30 hours and 725.30ml, respectively. The total post-surgical complication rate was 1.16%. The median hospital stay was 6.50 days. The pathological stages for T2 and T3 were 45.74% and 34.04%, respectively. The rate as positive surgical margins (PSMs) was 18.09% (pT2 and pT3; 6.38% and 9.57%). The angles between pubic bone and prostate gland (angle 1&2), were significantly associated with operative time and hospital stay, respectively (p<0.05). There was no correlation between the pelvimetry and positive surgical margin.

**Conclusions:**

The findings of the present study suggest that anthropometric measurements of the MRI are related to operative difficulties in ELRP. This study confirmed that MRI planning is the key to preventing complications in ELRP.

## INTRODUCTION

Prostate cancer (PCa) can be treated by radical prostatectomy (RP) which may provoke a troublesome side effect: urinary incontinence (UI). In addition, Lee CH (1) also suggest the likelihood of postoperative UI in patients undergoing LRP is markedly higher in those with larger intravesical prostatic protrusion. The keys to preventing complications of laparoscopic radical prostatectomy (LP) are meticulous preoperative evaluation of patients, magnetic resonance imaging (MRI) planning, and early diagnosis and management of complications (2). The extraperitoneal laparoscopic radical prostatectomy (ELRP) technique proved to be a safe and effective procedure in the treatment of prostate cancer when compared with the transperitoneal (TLRP) approach, with low morbidity (3).

There are few studies that have evaluated the influence of anthropometric measurements by MRI on perioperative outcomes in patients who underwent ELRP. In addition, there is controversy regarding the association between body habitus and perioperative outcomes of surgery, including bleeding, operative time (OT), and resection margins. Weimin (4) demonstrated that the poor view of the prostatic apex (VPA), protrusion of the prostate into the bladder, and high body mass index (BMI) were related to operative difficulties in ELRP. Also, Rue E (5) concluded that MRI before surgery did not provide a definite benefit to help the surgeon tailor LRP more accurately, according to the location and extent of the tumor, and thereby reduce the rate of positive surgical margins (PSMs). In addition, to our knowledge, no association has been reported between the curve distance, periprostatic plexus diameter and the outcomes of ELRP.

Thus, the aim of this study was to determine the association between anthropometric measurements of the MRI and perioperative outcomes on the OT, estimated blood loss (EBL), PSA, Gleason grade, pathological stage and PSMs in patients who underwent ELRP.

## MATERIALS AND METHODS

**Figure 1:**
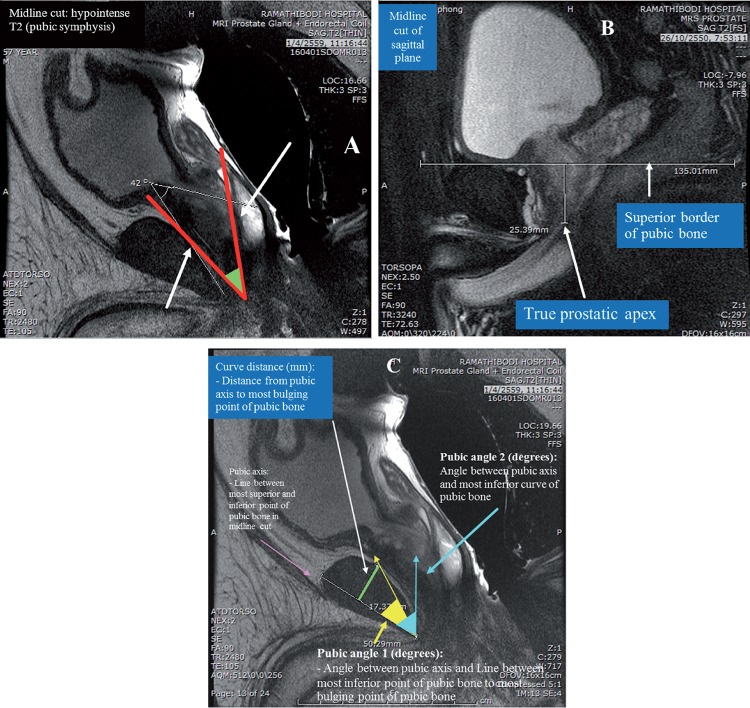
Anthropometric measurements by magnetic resonance imaging. (A) angle between the pubic bone and the prostate gland in midline cut: hypointense T2 (pubic symphysis); B) depth of prostatic apex; C) curve of public bone.

**Figure 2:**
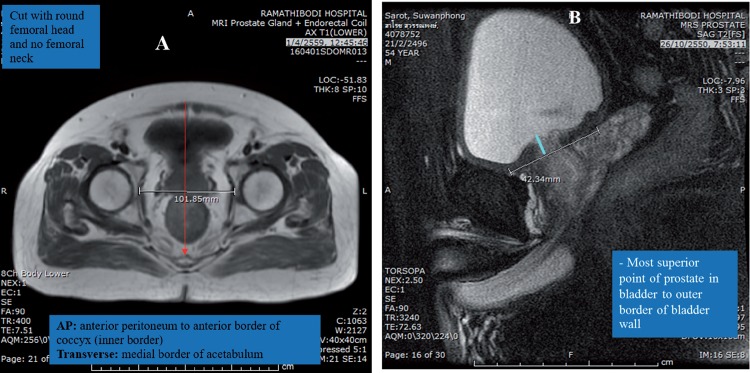
Anthropometric measurements by magnetic resonance imaging. (A) Work space transverse width (mm) on T1W image [AP: anterior peritoneum to anterior border of coccyx (inner border)] and Transverse: medial border of acetabulum; (B) Protrusion to bladder base (mm): most superior point of prostate in bladder to outer border of bladder wall.

From 2008 to 2014, 94 patients underwent ELRP for localized prostate cancer by the same experienced urologist (SP). In 86 patients, pelvic MR images were obtained at the time of prostate MRI before ELRP. For each patient, two clinically experienced radiologists (SA and TV), independently performed all the anthropometric measurements of MRI twice in each patient, in order to determine the mean value. The anthropometric measurements of MRI included prostatic size in volume by the ellipsoid formula [AP (cm) x Transverse (cm) x Vertical (cm) x 0.52], the angle between pubic bone and prostate (degree) (Figure-1A), depth of prostatic apex (mm) (Figure-1B), curve of pubic bone (Figure-1C) including curve distance (mm), pubic angles 1 (degrees) and 2 (degrees), abdominal wall thickness (mm), work space in AP (mm) and work space in transverse during surgery (mm) (Figure-2A), protrusion of the prostate into the bladder (mm) (Figure-2B), and retropubic fat and peri-prostatic plexus diameter.

The degree of the angle between the pubic bone and prostate gland was measured by drawing a line along the plane of the prostatic urethra and the line between the lowest points of the prostatic urethra to the most bulging point of the posterior cortex of the pubic bone. Curve distance (mm) was the perpendicular distance from pubic axis to the most bulging point of the posterior cortex of the pubic bone. Pubic angle 1 (degrees) was the angle between the pubic axis and the line between the most inferior point of the pubic bone to the most bulging point of the posterior cortex. Pubic angle 2 (degrees) was the angle between pubic axis and the most inferior curve of the pubic bone. The pubic axis was the line between the most superior and inferior points of the pubic bone in a midline cut. Workspace transverse width (mm) in AP was from the anterior peritoneum to the anterior border of the coccyx (inner border) and Transverse was the distance between the medial borders of the acetabulum. Protrusion to the bladder base (mm) was from the most superior point of the prostate in the bladder to the outer border of the bladder wall.

The institutional review board for research involving human subjects approved the retrospective analysis. We analyzed the associations between anthropometric measurements and patient demographics, including age, body mass index (BMI), preoperative prostate-specific antigen (PSA) level, pathologic stage, pathologic Gleason score, OT, EBL, surgical margin status and ‘30-day surgical-related complications’ defined as any complication rate.

### MRI Technique

Preoperative prostate MRI was performed on either a 1.5-T MR system (Signa HDxt, General Electric Medical System, USA) using endorectal and pelvic phase array coils or a 3.0-T MR system (Achieva, Philips Healthcare, USA) using a pelvic phase array coil. Immediately before the MR imaging examination, all patients underwent intravascular administration of 20mg of hyoscine-n-butylbromide to prevent peristalsis artifacts except when contraindicated.

All patients were imaged in the supine position. After the acquisition of localizing images, sagittal, coronal, axial thin-slice T2-weighted fast spin-echo (FSE) images through the prostate gland and seminal vesicles were obtained using the following parameters: TR range, 3,000-6,000 milliseconds (msec); TE, 104 milliseconds; echo-train length, 18; field of view (FOV), 16x16cm; section thickness, 3mm; interslice gap, 0mm; matrix 512x256; and number of excitations (NEX), 4. The transverse axial T1-weighted fast spine echo (FSE) images with a TR/TE of 400-600/10-15; matrix, 320x224; and all other parameters matched to the axial thin-slice T2W FSE sequence were obtained. The axial thin-slice T2-weighted images were used to calculate prostatic volume by Functool package post processing with the GE advantage workstation (GE Medical Systems).

Axial free-breathing DWI was performed using a single-shot echo-planar imaging technique with a TR of 3,000-6,000msec and a TE of 60-120msec; FOV, 18x18cm; section thickness, 5mm; interslice gap, 1mm; matrix 128x128; and NEX, 6. ADC values were obtained from the DWI sequences, which were performed with b values of 0.50 or 100, 800 or 1000, and 1500s/mm2. The ADC maps were generated by auto-calculation of the ADC value in each pixel of each slice.

Dynamic contrast enhanced MR imaging was performed by injecting a 0.1mmol/kg bolus of gadolinium-based contrast agent at a rate of 3ml/sec, followed by a 30ml saline flush at the same rate and serial T1W 3D images were obtained every 12 seconds through the entire prostate, using an MR-compatible automated injector (MedRad, USA). To allow acquisition of non-enhanced baseline images, the sequence and injection of the contrast agent were initiated simultaneously. A fast saturation-recovery TurboFLASH (fast low angle shot) sequence (TR 4.1msec, TE 1.9msec, flip angle 12°, matrix 256x192, FOV 200x240mm, slice thickness 5mm) was acquired. Total scan time was 5 minutes.

### Statistical analysis

Analysis of variance and comparison of proportions were used when indicated. Simple linear and logistic regression analyses were used to identify associative factors for OT, EBL, and PSMs. All tests were two-sided, with p≤0.05 considered statistically significant. Statistical analyses were conducted with use of Stata version 14.0 (Stata Corp, College Station, Texas, USA).

## RESULTS

The mean patient age was 69.61±8.30 years, and the patient's mean BMI was 24.86±3.29kg/m2. The mean preoperative serum PSA level was 16.31±21.20ng/ml. The median of OT and EBL was 5.23 (2.69; 6.86-4.18) hours and 600 (600; 900-300) ml, respectively. The only post-surgical complication was a single case (1.16%) of wound infection. The median hospital stay was 6.50 days (4.00; 9.00-5.00). The pathological stages, T2 and T3, were 45.74% and 34.04%, respectively. The rate of PSMs was 18.09% (17/94) (pT2 and pT3; 6.38% and 9.57%). Tables-1 and 2 demonstrates patient's characteristics and perioperative outcomes.

**Table 1:** Patient characteristics and perioperative outcomes.

No.	Variable	Mean±SD	Median (IQR: Q3-Q1)
1	Age (years)	69.61±8.30	71 (12.75: 75.75-63.00)
2	BMI (kg/m2)	24.86±3.29	24.79 (4.23: 26.71-22.48)
3	Serum PSA (ng/ml)	16.31±21.20	8.77 (11.77: 17.20-5.42)
4	Prostate volume (cc)	32.82±17.80	29.75 (13.95: 36.60-22.65)
5	Operative time (hours)	5.55±1.75	5.23 (2.69: 6.86-4.18)
6	Hospital stay (days)	8.24±4.06	4 (4: 9-5)
7	Estimated blood loss (ml)	725.30±539.50	600 (600: 900-300)

**SD: standard deviation; BMI: body mass index; PSA: prostate-specific antigen**

**Table - 2:** Patient pathological reports and positive surgical margins.

No.	Variable	N	Percentage (%)
1	Pathologic Gleason score		
	≤6	29	30.85
	7	48	51.06
	≥8	14	14.89
2	Pathologic stage		
	T1	7	7.45
	T2	42	44.68
	T3	31	32.98
	T4	2	2.13
3	Positive surgical margins	17	18.09

For the anthropometric measurements of MRI (Table-3), the mean prostatic volume was 32.72±17.41cc, the mean angle between the pubic bone and the prostate gland was 53.24±8.68 degrees; the mean depth of the prostatic apex was 29.00±6.10mm; the mean of curve distance was 14.28±2.70mm; the means of the pubic angles 1 and 2 were 23.10±3.81 and 48.70±10.11 degrees, respectively; and the mean abdominal wall thickness was 20.00±6.36mm. During surgery, the mean of workspace AP was 139.51±10.83mm and the mean of workspace transverse was 103.89±6.07mm; the mean of protrusion of the prostate into the bladder was 2.80±4.56mm and the means of retropubic fat and peri-prostatic plexus diameter were 3.20±2.02mm, and 3.30±0.79mm, respectively.

**Table 3:** Association among patient characteristics and prostate MRI and Using Univariate analysis using simple linear regression analysis.

Variable of MRI	Mean±SD	BMI		PSA		Operative Time (hr)		Blood loss (ml)		Hospital stay (days)		Positive Margin		Pathological Gleason score		Pathological stage	
		c.c.	P	c.c.	P	c.c.	P	c.c.	P	c.c.	P	c.c.	P	c.c.	P	c.c.	P
Prostate volume	32.717±17.41	0.02	0.409	0.01	0.942	0.00	0.996	-0.00	0.932	-0.02	0.551	-0.00	0.801	0.01	0.471	0.00	0.415
Angle between pubic bone and prostate (degree)	53.24±8.68	0.01	0.897	0.19	0.471	0.03	0.163	0.01	0.296	0.03	0.592	-0.01	0.165	-0.02	0.454	-0.00	0.702
Depth of prosatic apex (mm)	29.00±6.10	-0.01	0.821	0.30	0.425	0.00	0.982	0.02	0.124	-0.09	0.237	0.00	0.96	0.02	0.487	0.01	0.276
Curve distance (mm)	14.28±2.70	-0.14	0.272	-1.64	0.043	-0.03	0.663	-0.01	0.770	-0.30	0.061	0.01	0.707	0.01	0.921	0.04	0.171
Pubic angle 1 (degree)	23.10±3.81	-0.11	0.251	-0.90	0.123	-0.04	0.434	0.00	0.999	-0.29	0.015	0.00	0.847	0.05	0.253	0.02	0.329
Pubic angle 2 (degree)	48.70±10.11	0.01	0.804	-0.58	0.008	-0.04	0.025	0.01	0.203	-0.03	0.542	-0.00	0.875	-0.01	0.596	-0.01	0.480
Abd wall thickness (mm)	20.00±6.36	0.09	0.117	0.40	0.252	0.01	0.670	-0.00	0.901	0.12	0.087	0.01	0.408	0.00	0.971	0.01	0.264
Work space AP (mm)	139.51±10.83	0.03	0.403	-0.37	0.076	0.00	0.927	0.01	0.399	0.04	0.372	0.01	0.055	-0.01	0.770	-0.00	0.866
Work space transverse (mm)	103.89±6.07	0.02	0.780	-0.09	0.800	0.03	0.451	0.01	0.665	-0.07	0.361	-0.01	0.068	-0.01	0.769	0.00	0.980
Bladder protrusion (mm)	2.80±4.56	-0.01	0.856	0.97	0.049	0.02	0.606	0.02	0.177	-0.05	0.612	0.00	0.894	-0.06	0.114	0.02	0.360
Retropubic fat	3.20±2.02	-0.03	0.854	-2.12	0.057	0.00	0.979	-0.02	0.663	0.30	0.187	-0.01	0.540	-0.10	0.245	-0.05	0.177
Periprostatic plexus diameter (mm)	3.30 ±0.79	0.15	0.724	-0.18	0.951	0.37	0.119	0.07	0.516	-0.04	0.943	0.04	0.385	-0.16	0.475	0.02	0.840

**Abbreviation: BMI, body mass index; EBL, estimated blood loss; SD, standard deviation; c.c.; correlation coefficient**

According to simple linear regression analysis and the association between the anthropometric measurements of the MRI and perioperative outcomes of ELRP, the angles between pubic bone and prostate gland (angles 1 and 2), were significantly associated with operative time and hospital stay, respectively (p<0.05). Interestingly, pubic angle 2, curve distance and bladder protrusion were significantly associated with PSA (p<0.05). For multivariate analysis using simple linear regression analysis, pubic angle 2 was only significantly associated with PSA (p<0.05). There was no correlation between the pelvimetry and positive surgical margin. For the correlation among PSA, Gleason grade, pathological stage and the perioperative outcomes, PSA level was significantly associated with hospital stay (p<0.05) (Table-4).

**Table 4:** Association among PSA, Gleason grade and pathological stage and perioperative outcomes and using simple linear regression analysis.

Variable of MRI	Operative Time (hr)		Blood loss (ml)		Hospital stay (days)	
	c.c.	P	c.c.	P	c.c.	P
BMI	0.06	0.281	0.04	0.159	-0.05	0.734
PSA	0.02	0.035	0.01	0.100	0.01	0.535
Positive margin	0.42	0.390	0.13	0.563	1.82	0.116
Pathological Gleason score	-0.19	0.092	-0.06	0.258	-0.18	0.489
Pathological stage	-0.31	0.304	-0.09	0.545	-0.98	0.150

**BMI, body mass index; PSA, Prostate specific antigen; c.c.; correlation coefficient**

## DISCUSSION

ELRP allows direct access to the retropubic space, avoiding potential bowel injury, and it represents the technique that best replicates standard RP (6). There was no statistical difference from the transperitoneal techniques in OT, complication rates, or PSMs (7). Patients with a lowgrade impact of intravesical prostatic protrusion (IPP≤5mm) have significantly higher chances of recovering full continence (8). Multiparametric MRI positivity can independently predict biochemical recurrence after RP (9).

However, few studies have evaluated the influence of the anthropometric measurements of the prostate MRI on perioperative outcomes in patients who underwent ELRP. In 2010, Deok-Hyun et al. (6) determined the effect of pelvic arch interference and the depth of the pelvic cavity, as shown on preoperative MRI, on the performance of ELRP. The authors suggested that the depth of the pelvic cavity and prostate volume might increase surgical difficulty in patients undergoing ELRP by prolonging operative time. They measured the true conjugate diameter, the obstetric conjugate diameter, the difference between the true and obstetric diameters and the pelvic depth (the distance between the true conjugate and the prostate apex). Although the study was done in the same Asian population, all factors were different from the present study.

Our study demonstrated that the pubic angle 2 might increase surgical difficulty in patients undergoing ELRP by prolonging operative time. Prostate volume did not correlate with any anthropometric measurements of MRI. In our routine MRI of the prostate, the sagittal plane scanning technique does not include the sacral promontory due to the small field of view (FOV) in order to focus on the prostate gland. This prevented measurement of the true conjugate diameter. In this study, therefore, the pelvimetry was measured at the level of acetabulum on the axial image (both AP and transverse dimensions) to represent the working space for the urologists. However, this measurement was not correlated with operative time.

In the year 2010, Matikainen et al. reported the depth of prostatic apex is an independent predictor of positive apical margins at radical prostatectomy and confirmed MRI pelvimetry might allow for preoperative planning of open retropubic prostatectomy (RRP) or LRP (10) Interestingly, the pelvimetric measurements of the study were different from Deok-Hyun et al. method (6); the interspinous distance (ISD), the body width of the pelvis at the mid-femoral head level (BFW), the soft tissue width (SW) the apical depth (AD) and symphysis angle, In addition, these variables were developed by Hong et al. (11) as the pelvic dimension index (PDI; defined as ISD/AD), the bony width (BWI; defined as BFW/AD) and the SW index (SWI defined as SW/AD), The authors included each of PDI, AD, BWI and SWI as a measure of a “hostile” pelvis which is deep and narrow. The symphysis angle was defined as the angle axis of the symphysis pubis and the horizontal on the mid-sagittal T2-weighted sequence image.

Although the studies by Hong et al. (11) and Matikainen (10) were done in an Asian and the USA populations, respectively, these retrospective studies are limited by the small number of the population. The measurement of the pelvimetry in the present study was different from two previous reports, but very similar to the recently report by Weimin (4) that included the angle between the prostate and pubic bone and also the depth of prostate apex, in which, both parameters showed negative correlation with operative time. The surgery will be more difficult when the prostatic apex is located deep. Our study did not specifically mention a good and poor view of the prostatic apex (VPA), however, we developed two parameters i.e., curve distance and pubic angles that might influence laparoscopic techniques to approach the prostatic apex. The results showed that greater curve distances result in prolonged operative times. Since the present MRI technique did not demonstrate the perpendicular line from the promontory of the pelvis due to the narrow field of view (FOV), this study evaluated 4 different factors; first, curve distance, second, abdominal wall thickness, third, peri-prostatic plexus diameter and, fourth, the working space.

Weimin study (4) showed the surgical difficulty in patients undergoing ELRP related to different factors. The study demonstrated the view of the prostatic apex (VPA) was significantly associated with EBL (p=02), not operative time. In our study, however, there were no MR measurements correlated with EBL. Interestingly, the pubic angle 2 was not correlated with EBL, but was positively correlated with operative time. This may be explained by the fact that the surgeon needed to spend more time to control blood vessels to reduce bleeding. In addition, pubic angle 1 was significantly associated with hospital stay (p<0.05).

Weimin study (4) also reported that protrusion of the prostate into the bladder was significantly associated with positive resection margins (p=04) in multiple logistic regression analysis. Their positive surgical margin was very high (37%). The series of Matikainen (10) showed PSM rates of 10.4%, which were consistent with our literature. Our rate of PSMs of pT2 and pT3 was 6.38% and 9.57%, respectively. Moreover, the present study did not demonstrate any correlation between the pelvimetry and positive surgical margin, similar to Rud et al. results (5). High serum PSA, biopsy Gleason score of 7, low prostate volume, and interfascial NVB dissection were independently associated with side-specific PSMs after LRP, and should be considered during planning of the LRP surgical strategy (12). Moreover, our results confirmed PSA level was associated with prolonged LOS and confirmed the conclusion of Pearce report (13).

Although the study demonstrated that radiologists can work in a team with urologists, trying to obtain better results, there are several limitations to this study that should be noted. Firstly, this study is limited in that the data were collected retrospectively and the patients were not randomized for study. A high proportion of enrolled patients with more aggressive, intermediate and high risk tumors might have been included in our study. Secondarily, Shah et al. (14) indicated the effectiveness of 3.0T MRI and 1.5T endorectal MRI were similar in assessing diagnostic performance of cancer localization, extraprostatic extension, and seminal vesicle involvement; they demonstrated prostate anterior-posterior diameter measured was significantly shorter with 1.5T endorectal MRI than with 3.0T MRI. In addition, Albert et al. (15) also reported staging endorectal MRI should not be routinely used for treatment planning because it produces anatomic distortion. Therefore, further study is needed to clarify the differences between the two MRI systems in the association between the outcomes of ELRP and the anthropometric measurements of the prostate and illuminate whether MRI positivity can independently predict biochemical recurrence after LRP.

Finally, the most challenging laparoscopic surgery in Urology is ELRP (16). Birkmeyer et al. reported a variation in surgeon's technical skill based on peer-rated video-recording (17). Moreover, many studies reflect that during the learning curve a significant reduction in the average time to perform the urethral-bladder anastomosis, the estimated blood loss and the removal time of the urinary catheter have not caused any important complication while performing ELRP (3, 18). Our research design is from a Cross-Sectional analytic study with a 7-years series (from 2008-2014) and may have discrepancies when compared to other studies due to study population, surgeon experience and surgical procedures, similar to Ploussard et al. report (19).

## CONCLUSIONS

We believe there are benefits of performing MRI before ELRP to prevent complications of ELRP and suggest that anthropometric measurements of the MRI are related to operative difficulties. However, positive surgical margin was not influenced by the pelvimetry.
